# Efficient trajectory optimization for curved running using a 3D musculoskeletal model with implicit dynamics

**DOI:** 10.1038/s41598-020-73856-w

**Published:** 2020-10-19

**Authors:** Marlies Nitschke, Eva Dorschky, Dieter Heinrich, Heiko Schlarb, Bjoern M. Eskofier, Anne D. Koelewijn, Antonie J. van den Bogert

**Affiliations:** 1 Machine Learning and Data Analytics Lab, Department of Computer Science, Friedrich-Alexander-Universität Erlangen-Nürnberg (FAU), Erlangen, Germany; 2 Department of Sport Science, University of Innsbruck, Innsbruck, Austria; 3 adidas AG, Herzogenaurach, Germany; 4 Department of Mechanical Engineering, Cleveland State University, Cleveland, USA

**Keywords:** Computational biophysics, Musculoskeletal system, Biomedical engineering, Mechanical engineering, Applied mathematics, Biological physics

## Abstract

Trajectory optimization with musculoskeletal models can be used to reconstruct measured movements and to predict changes in movements in response to environmental changes. It enables an exhaustive analysis of joint angles, joint moments, ground reaction forces, and muscle forces, among others. However, its application is still limited to simplified problems in two dimensional space or straight motions. The simulation of movements with directional changes, e.g. curved running, requires detailed three dimensional models which lead to a high-dimensional solution space. We extended a full-body three dimensional musculoskeletal model to be specialized for running with directional changes. Model dynamics were implemented implicitly and trajectory optimization problems were solved with direct collocation to enable efficient computation. Standing, straight running, and curved running were simulated starting from a random initial guess to confirm the capabilities of our model and approach: efficacy, tracking and predictive power. Altogether the simulations required 1 h 17 min and corresponded well to the reference data. The prediction of curved running using straight running as tracking data revealed the necessity of avoiding interpenetration of body segments. In summary, the proposed formulation is able to efficiently predict a new motion task while preserving dynamic consistency. Hence, labor-intensive and thus costly experimental studies could be replaced by simulations for movement analysis and virtual product design.

## Introduction

In recent years, interest in musculoskeletal simulation to reconstruct and predict human movements has been growing^1^. Motion reconstruction based on captured data yields insight into further variables of interest, e.g. joint moments or muscle forces^2–7^. Furthermore, simulations can be applied to predict changes of kinematics as well as joint and muscle function in response to interventions or environmental changes. They can support decisions in orthopaedic surgeries^8,9^ and the design of prostheses^10,11^, exoskeletons^12^, or shoes^13^. Therefore, predictive simulations can replace time-consuming and expensive prototyping and experimental studies.

Commonly, biomechanical parameters are computed in a consecutive approach using inverse kinematics (IK), inverse dynamics (ID), and static optimization. This results in inconsistencies between kinematics and kinetics^14^. Additionally, each time step is analyzed separately in a discrete set of optimization problems rather than solving one optimization problem over time. Since either the motion or the forces are not simulated but prescribed, these methods cannot be applied to predict novel movements. These limitations can be overcome by solving an open-loop optimal control problem, also known as trajectory optimization. Movements are reconstructed or predicted by obtaining state and control trajectories from one single constrained non-linear optimization problem^7^. Alternatively, human motion can be simulated using a neuromuscular model with a controller based on reflexes^15,16^ or central pattern generators^17^.

Because of their relevance to daily activities and sports, it is important to also simulate movements with changes in direction additional to straight walking and running. It is also important that such movements can be optimized with respect to performance and/or injury risk, without having to collect human motion data from those movements. This will expand the use of existing data and avoid potentially risky human experiments. Potential applications are in knee injury prevention^18^, shoe performance in curved running^19^ or 3D controllers for exoskeletons and active prostheses^20^. Simulations of gait including turning were simulated with a reflex-based controller^16^, but reflex loops limit the space of possible control inputs. Trajectory optimizations find open-loop control trajectories without limiting the inputs, but have only been performed in two dimensional (2D) space^2,7,11,13,21–23^ or were restricted to forward motions^24–27^. A three dimensional (3D) model is needed to simulate movements with directional changes leading to a high-dimensional solution space of the optimal control problem. Therefore, effective numerical methods are necessary to put 3D optimal control simulations into application. Specifically, we need the capability to find solutions from an initial guess that is far from the solution, the solution should be found in a reasonable amount of time, and the solutions should exactly satisfy task requirements, such as a specified running speed and change of direction. The latter is of critical importance for sports applications.

Often, trajectory optimization problems were solved using an explicit formulation of the multibody dynamics leading to large computational costs^25–30^. The reconstruction of one cycle of walking or running took for example around 2 h for a 3D model with 21 degrees of freedom (DOFs) and 66 muscle tendon units (MTUs)^25^ using direct collocation with OpenSim’s explicit implementation of the dynamics^5,31^. This seems to be inefficient when taking into account that the initial guess was close to the simulation results since it was generated from human motion data of the same movement task^25^.

An implicit formulation of the model dynamics was proposed by Van den Bogert et al.^7^ for a 2D model to improve the numerical conditioning of the optimal control problem and thus reduced computational cost. They used direct collocation to solve tracking as well as predictive optimal control simulations. This approach was successfully applied to analyze loading asymmetry in transtibial amputee gait^11^ and the effect of midsole materials of shoes^13^. Recently, Falisse et al.^24^ used implicit dynamics to develop a framework for rapid simulations of a 3D model. They generated predictive simulations of walking and running with a full-body 3D model with 29 DOFs and 92 MTUs^32^ on average in 36 min. However, until now no movements with directional changes in 3D space were simulated.

The purpose of our work was to further extend the current state-of-the-art in trajectory optimization for musculoskeletal models by computational efficient simulations of movements with directional changes. To this end, we created a complex full-body 3D musculoskeletal model called “running model for motions in all directions”, short “runMaD”, adapted from Hamner et al.^32^. To reduce computational cost, dynamics were formulated implicitly, and derivatives were formulated analytically. We demonstrated the efficacy, the tracking capabilities, as well as the predictive power of the proposed trajectory optimization with implicitly formulated dynamics and direct collocation using three simulations: prediction of static standing, tracking of straight running, and prediction of curved running.

## Methods

In the following, we describe the developed musculoskeletal model, the general trajectory optimization approach and how we generated simulations of standing, straight running, and curved running.

### 3D musculoskeletal model

**Figure 1:**
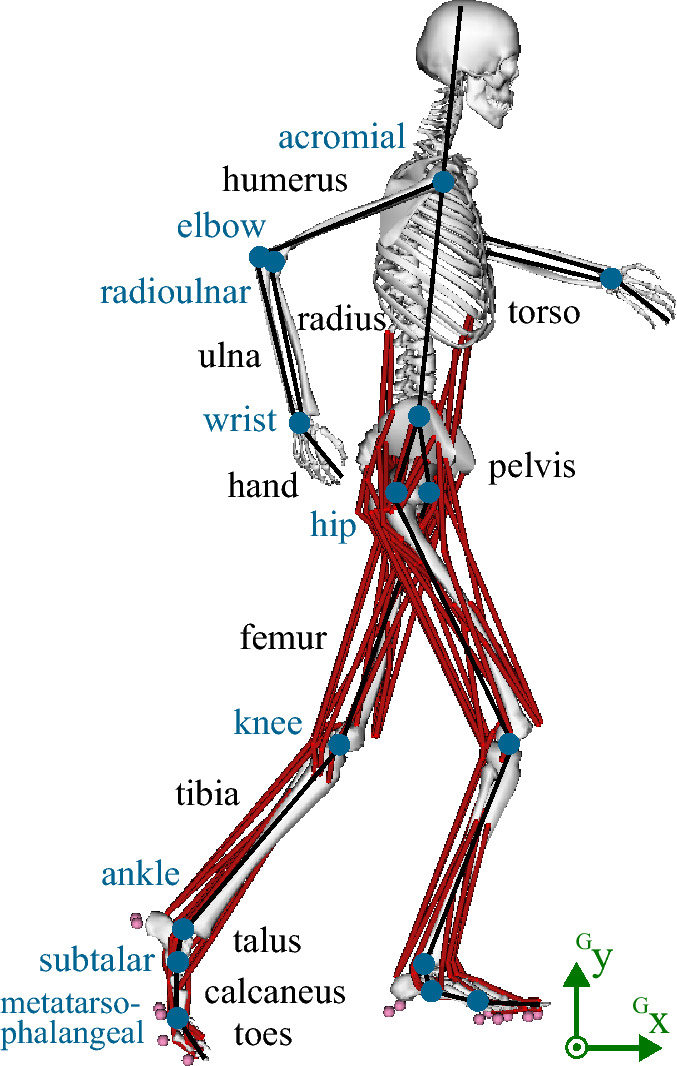
Musculoskeletal model “runMaD” with segments in black, joints in blue, ground contact points in pink, and the global coordinate system in green. The musculoskeletal model was visualized using OpenSim 4.0 (https://opensim.stanford.edu).

The proposed musculoskeletal model “runMaD” is a full-body 3D model with 33 DOFs, operated using 92 MTUs in the trunk and legs and 10 torque actuators in the arms (see Fig. 1). This model was adapted from the OpenSim model created by Hamner et al.^32^. The order of rotations in the pelvis was changed^33^ and the subtalar and metatarsophalangeal (mtp) joints were unlocked to simulate movements with directional changes. Ranges of motion were enlarged for knee flexion and pronation/supination angle at the elbow to fit the recorded motion. Muscular and segmental properties were taken from Hamner’s model. We refer to section S1 in the Supplementary Information for a detailed description of all model adaptations.

**Figure 2:**
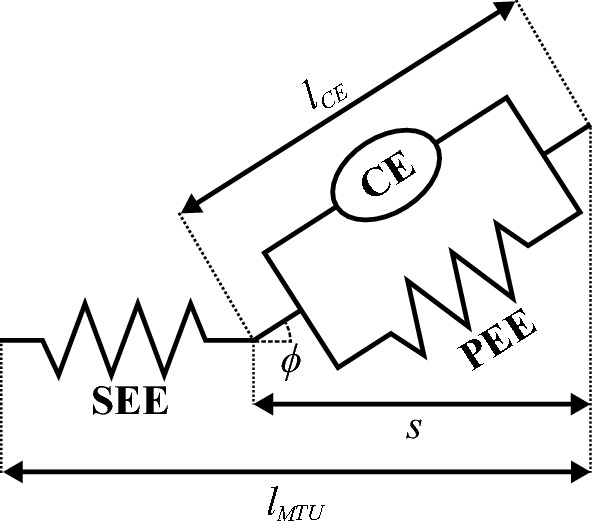
Hill-type muscle model^34^ with contractile element (CE), parallel elastic element (PEE), series elastic element (SEE), muscle-tendon length $l_{MTU}$, length of the CE $l_{CE}$, and pennation angle $\phi$. The state variable *s* represents the projection of $l_{CE}$ on the muscle line of action.

All muscles were modeled as three element Hill-type muscles with a contractile element (CE) with contraction and activation dynamics, a parallel elastic element (PEE), and a series elastic element (SEE) (Fig. 2)^34^. The dynamics were described implicitly for each muscle with respect to the activation $a$ and the state variable $s$, which was the projection of the CE length $l_{CE}$ on the muscle line of action^7^. The variable $s$ was used instead of $l_{CE}$ to avoid singularities with respect to the pennation angle $\phi$^7^. Muscle-tendon lengths were described as polynomial functions of joint angles, which were fitted using the muscle moment arm data of Hamner et al.^32^. In accordance with Falisse et al.^24^, polynomial functions were chosen since they have well-defined derivatives.

The model’s state vector was defined by $${\mathbf {x}}= ( \begin{array}{c} {\mathbf {q}}\quad {\dot{\mathbf {q}}}\quad {\mathbf {s}}\quad {\mathbf {a}}\end{array})^T$$, where $\mathbf {q}$ contained the DOFs, ${\dot{\mathbf {q}}}$ the derivatives of the DOFs, ${\mathbf {s}}$ the CE length state of all muscles, and $\mathbf {a}$ the activation state of all muscles. The control vector was defined as $${\mathbf {u}}= ( \begin{array}{c} {\mathbf {n}}_{\mathbf {e}}\quad {\mathbf {m}}\end{array})^T$$, where ${\mathbf {n}}_{\mathbf {e}}$ denoted the neural excitation of all muscles and $\mathbf {m}$ the arm actuation torque divided by 10 Nm for each of the DOFs in the arm (Eq. S15 in Supplementary Information). Dynamics of the musculoskeletal system were combined with a penetration-based ground contact model to describe the full dynamics of the model implicitly as function of the states $\mathbf {x}$, the state derivatives ${\dot{\mathbf {x}}}$, and the controls $\mathbf {u}$:

1 $$\begin{aligned} {\mathbf {f}}({\mathbf {x}}(t),{\dot{\mathbf {x}}}(t),{\mathbf {u}}(t))= {\mathbf {0}} . \end{aligned}$$

Details on the system dynamics are given in section S2 in the Supplementary Information.

### Trajectory optimization

Optimal control problems were formulated to generate movement simulations. The goal was to find a state trajectory ${\mathbf {x}}(t)$, a control trajectory ${\mathbf {u}}(t)$, and the duration of the simulated movement $T_{sim}$ such that the objective function $J({\mathbf {x}}(t),{\mathbf {u}}(t),T_{sim})$ was minimized with respect to the following constraints:

2 $$\begin{aligned}&{\mathbf {f}}({\mathbf {x}}(t),{\dot{\mathbf {x}}}(t),{\mathbf {u}}(t))\,= \,{\mathbf {0}}&(dynamic \,equilibrium) \end{aligned}$$

3 $$\begin{aligned}&{\mathbf {x}}_L(t) \,\le \,{\mathbf {x}}(t) \,\le \,{\mathbf {x}}_U(t)&(bounds \,on \,states) \end{aligned}$$

4 $$\begin{aligned}&{\mathbf {u}}_L(t) \,\le \,{\mathbf {u}}(t) \,\le \,{\mathbf {u}}_U(t)&(bounds \,on \,controls) \end{aligned}$$

5 $$\begin{aligned}&{\mathbf {x}}(T_{sim}) \,= \,{\mathbf {R}}_{\mathbf{per}}\,\mathbf {x}(0) \,+ {\mathbf{t}}_{\mathbf{per}}&(periodicity \,of \,states) \end{aligned}$$

6 $$\begin{aligned}&{\mathbf {u}}(T_{sim}) \,= \,{\mathbf {u}}(0)&(periodicity \,of \,controls) \end{aligned}$$

The periodicity constraint ensured that the state of the model at the end of the gait cycle, $T_{sim}$, was equal to the state at the beginning rotated in the horizontal plane with ${\mathbf {R}}_{\mathbf{per}}$ and shifted by horizontal translation ${\mathbf {t}}_{\mathbf{per}}$.

The objective was defined as weighted sum of a tracking term, a muscular effort term, a torque term, and a regularization term:

7 $$\begin{aligned} J({\mathbf {x}}(t),{\mathbf {u}}(t),T_{sim})\,= \,W_{Track}\,J_{track}\,+ \,W_{mus}\,J_{mus}\,+ \,W_{tor}\,J_{tor}\,+ \,W_{reg}\,J_{reg} . \end{aligned}$$

For tracking, the squared difference between simulated data $y_{sim}$ and the corresponding mean measured data $\mu _{y_{meas}}$ of multiple gait cycles was minimized for all time points $t$:

8 $$\begin{aligned} J_{track}\,= \,\frac{1}{T_{sim}} \int \limits _{0}^{T_{sim}} \sum \limits _{i=1}^{N_{track}} \,W_{Var,i}\,\left( \frac{y_{sim,i}(t) \,- \,\mu _{y_{meas,i}}(t) }{ \sigma _{y_{meas,i}}(t) } \right) ^2 \,dt . \end{aligned}$$

The squared difference was normalized by the variance of measured data $\sigma _{y_{meas}}^2$ to make it dimensionless. The variance $\sigma _{y_{meas}}^2$ was adapted to be at least 10 % of the mean of the variance to avoid division by small numbers. This is for example necessary for the variance of the ground reaction force (GRF) during swing phase. Furthermore, the terms were normalized by the duration of the simulation $T_{sim}$ and weighted with $W_{Var,i}$ for each signal variable *i*. Volume-weighted and cubed neural excitation $n_e$ was minimized for each of the $N_{mus}$ muscles to reduce muscular effort and to solve the muscle redundancy problem:

9 $$\begin{aligned} J_{mus}\,= \,\frac{1}{T_{sim}\,N_{mus} \left\Vert \left( {\begin{smallmatrix} v_x\\ v_z\end{smallmatrix}}\right) \right\Vert ^3 } \int \limits _{0}^{T_{sim}} \sum \limits _{i=1}^{N_{mus}} \left( \frac{ F_{ISO,i}\,l_{CE,opt,i}}{ \sum \limits _{i=1}^{N_{mus}} F_{ISO,i}\,l_{CE,opt,i}} \,n_{e,i}(t)^3 \right) \,dt , \end{aligned}$$

where the ratio of muscle volume was computed with the maximum isometric force $F_{ISO}$ and the optimal length of the CE $l_{CE,opt}$. The muscle effort was divided by the cubic norm of horizontal translation speeds $$\left\Vert \left( {\begin{smallmatrix} v_x\\ v_z\end{smallmatrix}}\right) \right\Vert ^3$$ to compensate for different running speeds. It was shown previously that muscle activation is linear to movement speed^35,36^. Besides the control of the muscles, the torque controls $m_i$ actuating the arms were minimized:

10 $$\begin{aligned} J_{tor}\,= \,\frac{1}{T_{sim}\,N_{tor}} \int \limits _{0}^{T_{sim}} \sum \limits _{i=1}^{N_{tor}} \,m_i(t)^2 \,dt . \end{aligned}$$

Finally, a small regularization term was added to enhance convergence by minimizing the derivatives of the states ${\mathbf {x}}$ and controls $\mathbf {u}$:

11 $$\begin{aligned} J_{reg}\,= \,\frac{1}{T_{sim}\,(N_{states}\,+ \,N_{controls}) } \int \limits _{0}^{T_{sim}} \left( \sum \limits _{i=1}^{N_{states}} \,{\dot{x}}_i(t)^2 \,+ \,\sum \limits _{i=1}^{N_{controls}} \,{\dot{u}}_i(t)^2 \,\right) \,dt . \end{aligned}$$

We used regularization for both states and controls since we found that this yields the lowest number of iterations without losing simulation accuracy.

### Simulations

**Figure 3:**
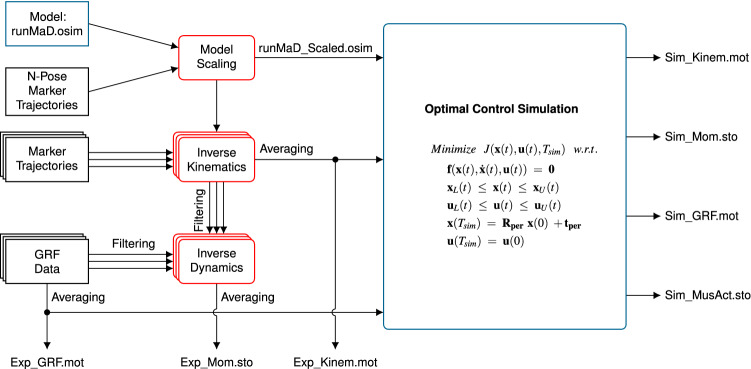
Processing pipeline. Work presented in this paper is highlighted in blue and OpenSim applications in red. The unscaled and scaled model, the experimental data, as well as the simulation results are provided in OpenSim file formats in the electronic supplementary material and at https://simtk.org/projects/runmad.

We performed three simulations: prediction of static standing, tracking of straight running, and prediction of curved running. In the following, we describe the data acquisition, the optimal control problems of the three simulations, and the details of implementation and solution process. An overview of the pipeline is given in Fig. 3.

#### Experimental data

We recorded straight and curved running of a male subject (92 kg, 1.95 m) using 42 reflective markers, 16 infrared cameras (Vicon MX, Oxford, UK), and two force plates (Kistler Instruments Corp, Winterthur, CH) for tracking and as reference. The sampling frequency was set to 200 Hz and 1000 Hz, respectively. Straight running was performed at a speed of $v_x= {4.0}\,\hbox {ms}^{-1}$ and $v_z= {0}$. Curved running was performed in a circle with radius $r= {3.7}\,\hbox {m}$ at a norm horizontal speed of $$\left\Vert \left( {\begin{smallmatrix} v_x\\ v_z\end{smallmatrix}}\right) \right\Vert = {2.7}\,\hbox {ms}^{-1}$$. The API of OpenSim 4.0^5^ was called within MATLAB (Mathwork, Natick, MA, USA) to scale the generic model using marker trajectories in neutral pose with arms besides the body (N-pose) and to compute IK and ID. For ID, joint angles were filtered within OpenSim with a 3rd order dual-pass low-pass Butterworth filter with a cut-off frequency set to 15 Hz^37^. The GRFs were filtered with the same filter to avoid artifacts in the computed joint moments^38^. After processing, single gait cycles were extracted from right to right heel strike using the minimum of the right heel marker. The mean and standard deviation (SD) of 12 gait cycles were computed for straight and curved running after linearly interpolating to the number of samples of the shortest cycles. The subject gave informed consent prior to participation. The study was approved by the ethical committee of the Friedrich-Alexander-Universität Erlangen-Nürnberg (Re.-No. 106_13 B). All methods were carried out in accordance with relevant guidelines and regulations.

#### Standing

The goal of the prediction of static standing was to find a neutral pose of the model in equilibrium without data tracking. As this simulation was independent of time, ${\dot{\mathbf {x}}}$ was set to $\mathbf {0}$ and no periodicity constraints (Eqs. 5 and 6) were used. The weights of the objective terms were chosen empirically for all simulations. For standing, $W_{mus}= W_{tor}= 1$ was set. All bounds of $\mathbf {x}$ and $\mathbf {u}$ are provided in Table S2 in the Supplementary Information. The simulation was solved 50 times with different random initial guesses for states $\mathbf {x}$ and controls $\mathbf {u}$ to reduce the likelihood of ending up in a local minimum. The result with the lowest objective was chosen as solution.

#### Straight running

Straight running was reconstructed by tracking straight running data. All joint angles, the global orientation of the pelvis and the GRFs of both feet in all directions were tracked (Eq. 8) similar to our approach in 2D^7^. A weighted arithmetic mean was used to balance the influence between joint angle tracking and GRF tracking independently of the number of signals: $W_{Var,i}\,= \,\frac{W_{ang/GRF}}{N_{Ang}\,W_{ang}\,+ \,N_{GRF}\,W_{GRF}}$ with $W_{ang}=1$ and $W_{GRF}=5$ and the numbers of tracked signals $N_{Ang}$ and $N_{GRF}$. The running speed used for weighting in the muscular effort term (Eq. 9) was computed from the tracking data. The weights of the objective terms were set to $W_{Track}=1$, $W_{mus}=10^3$, and $W_{tor}=1$ such that after optimization the weighted objectives of tracking and effort were of same scale. The weight of the regularization term was small.

In the straight running simulation, the periodicity of the gait cycle was ensured by allowing only translation in the horizontal plane with prescribed running speeds (Eq. 5). Hence, ${\mathbf {R}}_{\mathbf{per}}$ in Eq. (5) was the identity matrix and only a translation for the global pelvis position was applied:

12 $$\begin{aligned} \begin{pmatrix}q_{pel\_tx}(T_{sim}) \\ q_{pel\_ty}(T_{sim}) \\ q_{pel\_tz}(T_{sim})\end{pmatrix} \,= \,\begin{pmatrix}q_{pel\_tx}(0) \\ q_{pel\_ty}(0) \\ q_{pel\_tz}(0)\end{pmatrix} \,+ \,\begin{pmatrix}\,v_x\,T_{sim}\\ 0 \\ \,v_z\,T_{sim}\end{pmatrix} . \end{aligned}$$

Additionally, the states $\mathbf {x}$ and controls $\mathbf {u}$ were limited by lower and upper bounds (Eqs. 3 and 4). To define a global start position of the motion, the pelvis position at the first node was fixed to $q_{pel\_tx}[0] = q_{pel\_tz}[0] = 0$. The standing solution was used as initial guess.

#### Curved running

Curved running was predicted by tracking straight running data and constraining the model to run in a circle. Only joint angles and vertical GRFs were tracked to allow a circular motion (Eq. 8). The norm horizontal speed was obtained from the reference data of curved running to weight the muscular effort term (Eq. 9). In contrast to straight running, $W_{mus}$ and $W_{tor}$ were increased by factor 10 to allow more deviation from the tracking data.

With help of the periodicity constraint (Eq. 5), we ensured that the model ran counterclockwise in a circle around the y-axis so that the left leg was on the inside. The circle was centered at $$\left( {\begin{smallmatrix} q_{pel\_tx}\\ q_{pel\_tz}\end{smallmatrix}}\right) = \left( {\begin{smallmatrix} 0 \\ 0 \end{smallmatrix}}\right)$$ with central angle $\theta$. All entries in the state vector $\mathbf {x}$ were constrained to be equal for $t=0$ and $t= T_{sim}$ except for the global pelvis position and rotation:

13 $$\begin{aligned} \begin{pmatrix} q_{pel\_tx}(T_{sim}) \\ q_{pel\_ty}(T_{sim}) \\ q_{pel\_tz}(T_{sim}) \\ q_{pel\_rot}(T_{sim}) \end{pmatrix} \,= \,\begin{pmatrix} cos(\theta ) &{} 0 &{} sin(\theta ) &{} 0\\ 0 &{} 1 &{} 0 &{} 0\\ -sin(\theta ) &{} 0 &{} cos(\theta ) &{} 0\\ 0 &{} 0 &{} 0 &{} 1 \end{pmatrix} \,\begin{pmatrix} q_{pel\_tx}(0)\\ q_{pel\_ty}(0)\\ q_{pel\_tz}(0)\\ q_{pel\_rot}(0) \end{pmatrix} \,+ \,\begin{pmatrix} 0 \\ 0 \\ 0 \\ \theta \end{pmatrix} . \end{aligned}$$

The norm horizontal speed and the radius of the measured curved running were used to obtain the central angle:

14 $$\begin{aligned} \theta \,= \,2 \,\arcsin \left( \frac{\left\Vert \left( {\begin{smallmatrix} v_x\\ v_z\end{smallmatrix}}\right) \right\Vert \,T_{sim}}{2 \,r} \right) . \end{aligned}$$

To define a global start position of the motion, the pelvis position at the first node was fixed to $q_{pel\_tx}[0] = -r$ and $q_{pel\_tz}[0] = 0$. The straight running solution was used as initial guess.

#### Implementation and solution process

The implicit multibody dynamics of the skeletal model were derived using Autolev (Symbolic Dynamics Inc., Sunnyvale, CA, USA) including Jacobian matrices generated by symbolic differentiation. Muscle dynamics and the ground contact model were implemented in C. All dynamic equations were compiled as MEX-functions in MATLAB. The objectives and the task constraints as well as their analytic derivatives were coded in MATLAB.

The optimal control problems were solved for the scaled musculoskeletal model using direct collocation and backward Euler discretization. One collocation node was used for the static standing simulation. 50 collocation nodes were chosen for the running simulations since we found in preliminary simulations that 50, 100 and 200 collocation nodes yielded similar results. Running data was linearly interpolated to 50 samples. The non-linear optimization problems were solved with IPOPT 3.12.3^39^. Settings were adapted to terminate the optimization at a tolerance of 10^−5^, a constraint violation tolerance of 10^−3^, and a complementary tolerance of 10^−3^. All optimizations were run on one core of a workstation with a 3.2 GHz Xeon E5-1660v4 processor.

## Results

In total, 50 predictive simulations of static standing using different random initial guesses, one tracking simulation of straight running, and one predictive simulation of curved running were solved. CPU times and iterations required for optimization are summarized in Table 1. The entire process from generating standing from random initial guesses to the prediction of curved running took 1 h 17 min 28 s. The tracking of straight running using standing as initial guess required more iterations than the prediction of curved running using straight running as initial guess since standing is quite a different task than running.

**Table 1:** Solver performance for the simulations of standing, straight running, and curved running.

		CPU time in mm:ss	Iterations
**Standing**	Result	00:10	355
	Mean	00:14	513
	Sum	11:26	25,669
Straight running		46:22	1,065
Curved running		19:40	715

For standing, the CPU times and number of iterations are listed for the chosen result which had the lowest objective of the 50 simulations. Additionally, the mean and sum for all 50 standing simulations is given.

**Figure 4:**
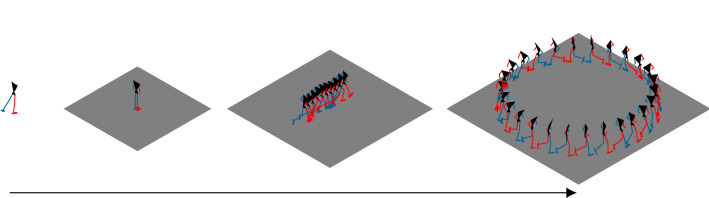
Stick figures showing the simulation process. From left to right: Initial guess used for standing, result of standing, result of straight running, and result of curved running. For visualization, every fifth and every 25th node was plotted for straight and curved running, respectively, and the curved running result was extended to fill a whole circle.

**Figure 5:**
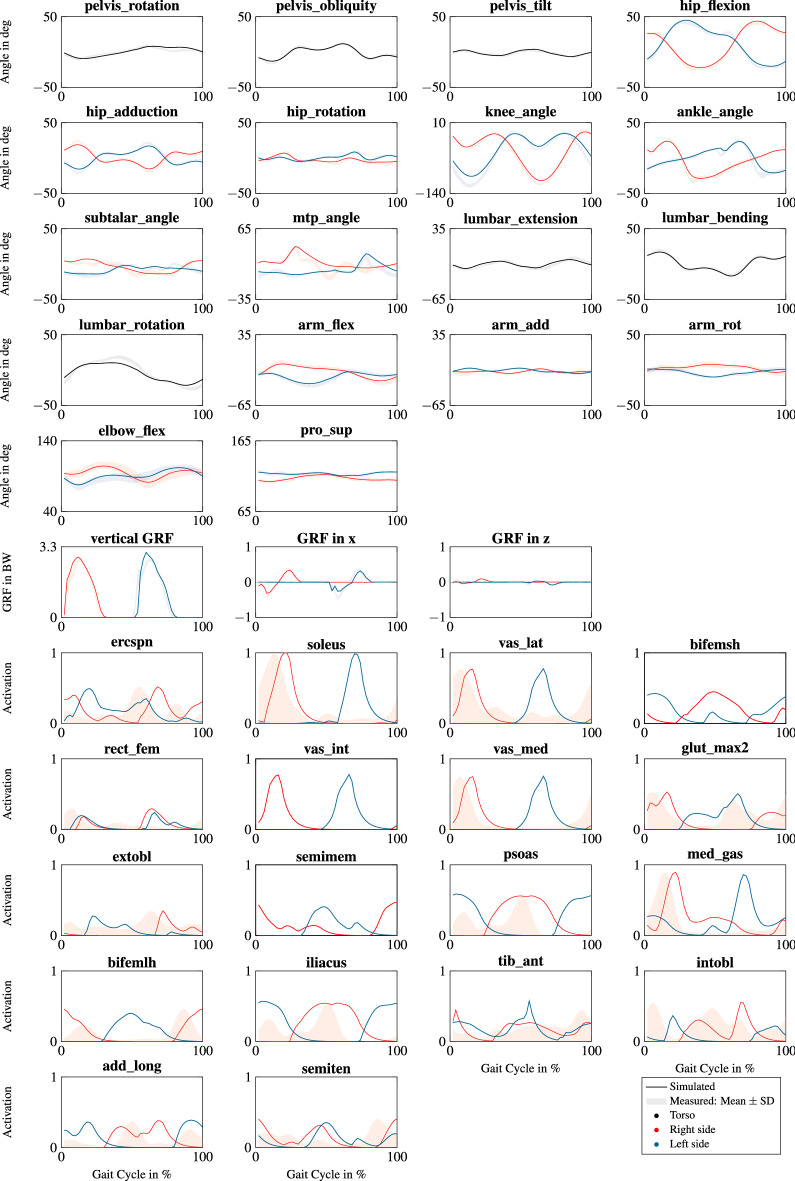
Joint angles, GRFs, and muscle activations of the 18 largest muscles of the straight running simulation at 4.0 ms^−1^. GRFs are scaled to body weight (BW) and muscle activations are normalized to the peak activation of straight running. The degrees of freedom (DOFs) and muscles are named according to their definition in the model file runMaD.osim. An overview of all muscles is provided in Table S1 in the Supplementary Information. Black, red, and blue solid lines indicate the simulated variables of the torso, the right side, and left side, respectively. For GRFs and joint angles, shaded areas show mean ± standard deviation (SD) of the measured gait cycles of straight running. For muscles, shaded red areas show mean electromyography (EMG) data of the right side for running at 3.3 ms^−1^ reported by Cappellini et al.^40^ normalized to the maximum of the simulated activations of each muscle.

The results corresponded to upright standing and natural running motion (Fig. 4 and videos in the electronic supplementary material). Joint angles and GRFs of straight running were close to the reference data of straight running, since the difference was generally less than one SD (Fig. 5). However, simulated knee flexion was smaller during the swing phase compared to the reference data. The difference between the right and left subtalar angle was well represented in the simulation of straight running even though subtalar and mtp angles deviated from the reference data more than one SD. The differences in GRFs were larger than one SD between 20% and 40% of the gait cycle. The estimated muscle activation patterns of the 18 largest muscles were similar to the electromyography (EMG) measurements reported by Cappellini et al.^40^ for straight running (Fig. 5).

**Figure 6:**
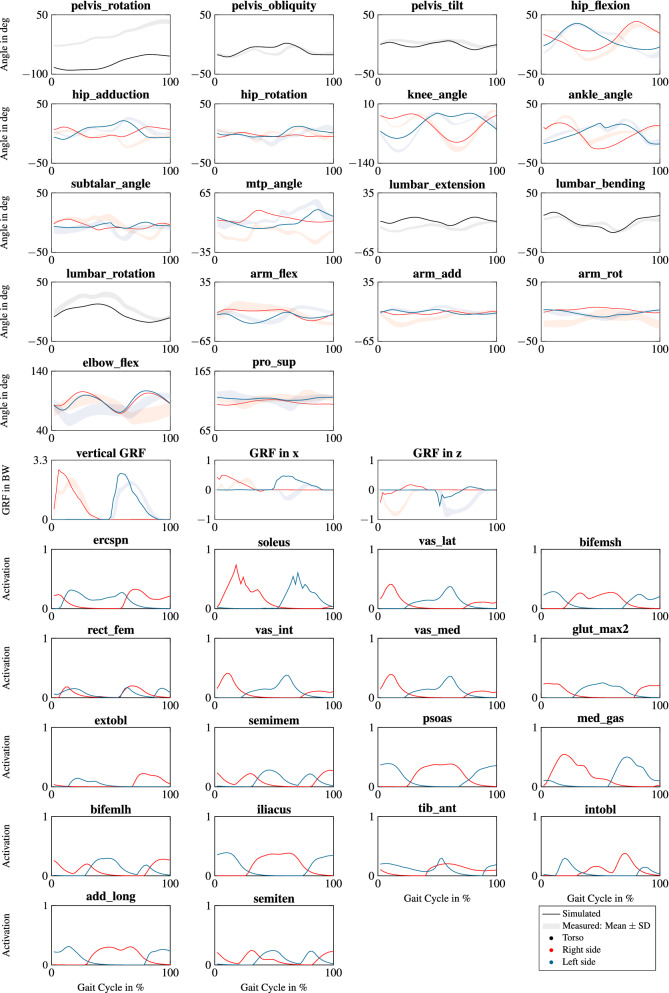
Joint angles, GRFs, and muscle activations of the 18 largest muscles of the curved running simulation at 2.7 ms^−1^. GRFs are scaled to body weight (BW) and muscle activations are normalized to the peak activation of straight running. The degrees of freedom (DOFs) and muscles are named according to their definition in the model file runMaD.osim. An overview of all muscles is provided in Table S1 in the Supplementary Information. Black, red, and blue solid lines indicate the simulated variables of the torso, the right side, and left side, respectively. Shaded areas show mean ± standard deviation (SD) of the measured gait cycles of curved running.

Predicted joint angles and GRFs of curved running matched the reference data of curved running but were not as similar as for the tracking simulation (Fig. 6). In particular, the ranges of motion were underestimated in the hip and knee flexion, knee flexion was smaller during stance, and maximum vertical GRFs were overestimated. The pelvis rotation and the horizontal GRFs cannot be compared directly for curved running since the global frames of simulation and reference were not aligned but rotated around the vertical axis. Muscle activations for curved running were similar to straight running but of less amplitude and less symmetric (Fig. 6). The model and data of the simulated and measured movements is provided in OpenSim file formats in the electronic supplementary material and at https://simtk.org/projects/runmad.

## Discussion

In this work, we presented a 3D full-body musculoskeletal model adapted to running with directional changes and an implicit formulation of its dynamics for efficient trajectory optimization using direct collocation. We generated a predictive simulation of standing, a tracking simulation of straight running, and a predictive simulation with directional change, more precisely curved running, to demonstrate the efficacy, the tracking capabilities, and the predictive power of the approach.

Computational efficiencies must be compared with caution due to influences of computational power, implementation, the initial guess, and the choice of the numerical solution method, e.g. direct collocation^7,25,26^, multiple shooting^41,42^, and simulated annealing^27^. Nevertheless, it can be concluded that the CPU times of approximately 46 min and 20 min for straight and curved running, respectively, were small compared to simulations with explicit dynamics^25,26^. We were able to solve tracking simulations of straight running faster than Lin and Pandy^25^ and Lin et al.^26^, while starting from an initial guess that was constructed without IK, static optimization, or computed muscle control (CMC). Furthermore, our model “runMaD” had a higher complexity resulting in approximately 20 % more unknowns, i.e. states and controls, per collocation node. The CPU times were similar to Falisse et al.^24^, who simulated straight running, confirming the advantages of implicit formulation of model dynamics. Falisse et al.^24^ used algorithmic derivatives whereas we implemented them analytically. Future studies are needed to compare the efficiency of algorithmic and analytical derivatives in optimal control simulations. Previously, it was shown that using algorithmic derivatives is faster than finite differences which are used by OpenSim^43^. Machine learning approaches were recently investigated in the field of computer graphics to speed up musculoskeletal simulation^44,45^. Jiang et al.^44^ learned a mapping from muscle-actuation space to joint-actuation space which would have to be retrained if model parameters are changing. Lee et al.^45^ used a two-stage deep reinforcement learning approach to simulate a full-body 3D musculoskeletal model. However, muscle activation was not included in the reward of the trajectory mimicking which might lead to non-optimal use of the muscles.

In all three simulations, kinematics were natural and matched the reference data. However, smaller knee flexion during the swing phase resulted in a lower foot clearance in both running simulations (Figs. 5 and 6). This movement pattern might be more energy efficient since increased foot clearance requires more effort. Subtalar and mtp angles deviated from the reference data and seemed to compensate each other’s error (Figs. 5 and 6). The reference data might be erroneous since the subject wore shoes and the foot segments are small which increases sensitivity to errors in marker positions and soft tissue artifacts. Additionally, simplifications in the foot model likely caused inaccuracies in the simulation. Although our proposed model is allowing subtalar and mtp motion in contrast to the model of Hamner et al.^32^, our foot model does also not reflect the fine foot structures. For a detailed analysis of foot motion, a finite element model of the foot, like developed by Akrami et al.^46^, could be incorporated into the musculoskeletal model. Instead of data tracking, movements could be reconstructed by constraining the movement path^25,26,42^. Nevertheless, the bounds cannot be exceeded and are difficult to choose especially if a change in motion should be predicted.

In the predictive simulation of curved running, interpenetration of the legs occurred to reduce muscular effort (see video in electronic supplementary material). As a result of this, kinematics deviated slightly from reference data, i.e. ranges of motion were underestimated in the hip and knee flexion (Fig. 6). Interpenetration could be avoided by prescribing a minimal distance between joint origins^24^ which would however require prior knowledge about the motion path. Alternatively, a constraint or an error term could be added to prevent intersection of segments similar to what was done in computer graphics^47^. In both cases, the use of a stochastic environment^48^ would be beneficial to avoid segments moving close to each other. Similarly, the predictive simulation was lacking knee flexion during stance since it does not account for uncertainties while minimizing effort^24,49^.

Even though GRFs of straight running were tracked well, impact of the initial contact was not distinct due to a fast progression towards the forefoot (Fig. 5). This was caused by the relatively coarse sampling with 50 nodes. Additionally, the simple foot and contact model might have influenced especially the impact. For curved running, maximum vertical GRFs were overestimated compared to the reference data (Fig. 6) probably due to the interpenetration of the legs. Nevertheless, the outer leg, i.e. the right leg, showed a higher maximum vertical GRF compared to the inner leg in agreement with the reference data.

Joint moments were smoother in the simulation compared to the ID since the effort term has a smoothing effect (Figs. S1 and S2 in the Supplementary Information). ID depends on filtering of joint kinematics and GRFs^38^ whereas trajectory optimization benefits from a physics-based filtering. The mtp moments cannot be compared to the reference until late stance phase since GRFs were applied to the calcaneus in ID whereas we simulated ground contact at the calcaneus and toe segment. However, the mismatch of simulated and measured subtalar and mtp angles, contributed to the deviation in joint moments.

In contrast to static optimization, muscle activations and controls were computed while accounting for muscle and tendon dynamics. This is especially important for the analysis of fast movements, like running^50^. The simulated muscle activations for straight running followed mainly the patterns of EMG measurements^40^ (Fig. 5) despite a higher velocity (4.0 ms^−1^ vs. approximately 3.3 ms^−1^). In comparison to straight running, peak muscle activations were smaller for curved running since running velocity was smaller (4.0 ms^−1^ vs. 2.7 ms^−1^) (Fig. 6). Furthermore, muscle activations were less symmetric due to the asymmetric movement task. We were able to predict curved running at 2.7 ms^−1^ while using tracking data of straight running at a different velocity of 4.0 ms^−1^. This confirms the predictive power of the trajectory optimization, because the target and tracking velocity did not have to match.

Three general limitations of trajectory optimization have to be mentioned. First, it cannot be ensured that the global optimum of the optimal control problem was found. In the standing simulation, we used multiple initial guesses to minimize the risk of ending in a local optimum. Second, reported kinematics and kinetics were affected by the objective function and thus by the choice of weights of the objective terms. GRFs were weighted more in comparison to joint angles since the GRFs were the only tracked signals containing information about the forces within the body. For higher tracking weight $W_{Track}$ in the straight running simulation, GRFs and joint angles were tracked better. However, this came at the cost of non-smooth activation signals. The signals contained alternating phases of activation and deactivation to allow the joint angles to closely match the data. When the same weights of the straight running simulation were used for the curved running simulation, i.e. when effort weight was decreased, the result was closer to the tracking data resulting in higher knee flexion, higher knee moment and higher activation in knee extensors during stance. However, the other variables were predicted worse. Weights in the objective function could be obtained from data using inverse optimal control instead of selecting them empirically^41,51^. Third, it is not yet known which energy measure is minimized in human walking. Several studies proposed that metabolic energy is minimized^52–54^, while others hypothesized that it is more likely that muscular effort, which is related to activation and thus neural excitation, is minimized in human gait^22,49,55^. Since the actual movement objective is unknown, we included tracking. The simulation is still predictive because a new motion task was simulated based on another one. A different option is to manually tune weightings of energy measures to predict walking and running^24^. However, instead of data, this requires expert input.

In conclusion, we presented a comprehensive 3D full-body musculoskeletal model modified for biomechanical analysis of running with directional change, i.e. curved running. Model dynamics were formulated implicitly resulting in computational efficient simulations. The efficiency makes large scale inverse optimal control studies or sensitivity studies actually feasible. Furthermore, virtual product design^11,13^ would considerably benefit. Predicted kinematics and kinetics confirmed the predictive power of the proposed approach and were very promising but limited by the fact that the true objective of human motion is still unknown. For this reason, this work might be an important step towards efficient and biomechanical accurate predictive simulations of movements including directional changes.

## Supplementary information

Supplementary information 1.

Supplementary information 2.

Supplementary information 3.

Supplementary Dataset.
