# Supplementary material for: Efficient trajectory optimization for curved running using a 3D musculoskeletal model with implicit dynamics
Source: Sci Rep. 2020 Oct 19;10:17655. doi: 10.1038/s41598-020-73856-w (PMC7573630; doi:10.1038/s41598-020-73856-w)
Supplement: Supplementary file 1 — Supplementary information 1. [file 41598_2020_73856_MOESM1_ESM.pdf]

# Supplementary Information

# Efficient trajectory optimization for curved running using a 3D musculoskeletal model with implicit dynamics

**Marlies Nitschke[1,*], Eva Dorschky[1], Dieter Heinrich[2], Heiko Schlarb[3], Bjoern M. Eskofier[1], Anne D. Koelewijn[1,4], and Antonie J. van den Bogert[4]**

[1]Machine Learning and Data Analytics Lab, Department of Computer Science, Friedrich-Alexander-Universität Erlangen-Nürnberg (FAU), Erlangen, Germany
[2]Department of Sport Science, University of Innsbruck, Innsbruck, Austria
[3]adidas AG, Herzogenaurach, Germany
[4]Department of Mechanical Engineering, Cleveland State University, Cleveland, USA
[*]marlies.nitschke@fau.de

## S1 Model Adaptations

The proposed “running model for motions in all directions”, short “runMaD”, was adapted from a model proposed by Hamner et al.[1] in order to simulate running with directional changes. We changed the sequence of pelvis rotations from tilt (z), list (x), rotation (y) to rotation (y), obliquity (x), tilt (z) (see Fig. 1 for the global coordinate system). The updated rotation sequence is in agreement with clinical understanding[2]. Hence, results are interpretable in clinical analysis independently of the movement direction. Furthermore, this sequence simplifies the specification of directional tasks in simulations, e.g. in the presented curved running simulation.

Pronation and supination of the foot was enabled by unlocking the subtalar joint. The metatarsophalangeal (mtp) joint was also unlocked for roll over of the foot. For both joints, a range of motion from $-90°$ to $90°$ was allowed. In order to fit the recorded data of fast running, the upper limit of knee flexion was increased from $120°$ to $160°$. Additionally, the range of motion of the pronation/supination angle at the elbow was enlarged from $[0°, 90°]$ to $[0°, 150°]$ and the default pronation/supination angle was set to $90°$ such that the palms were pointing towards the body for zero torque. The default elbow flexion was set to $5°$ to be within the range of motion.

## S2 System Dynamics

The system dynamics were described implicitly as a function $\mathbf{f}()$ of the states $\mathbf{x}$, the state derivatives $\dot{\mathbf{x}}$, and the controls $\mathbf{u}$ (Eq. 1). The function $\mathbf{f}()$ contained

- identities $\dot{\mathbf{q}} - \frac{d\mathbf{q}}{dt} = \mathbf{0}$ for each degree of freedom (DOF),
- multibody dynamics for each DOF (Eq. S1),
- activation dynamics for each muscle tendon unit (MTU) (Eq. S2), and
- contraction dynamics for each MTU (Eq. S3).

### S2.1 Multibody Dynamics

The multibody dynamics were defined as follows:

$$\mathbf{M}(\mathbf{q})\,\ddot{\mathbf{q}} + \mathbf{C}(\mathbf{q},\dot{\mathbf{q}})\,\dot{\mathbf{q}} + \mathbf{G}(\mathbf{q}) - \mathbf{J_c}^T\,\mathbf{F_c} - \boldsymbol{\tau} = \mathbf{0}, \tag{S1}$$

where $\mathbf{q}$ contained the global position, the global orientation, and the joint angles, $\dot{\mathbf{q}}$ contained the global velocities and joint angular velocities, $\mathbf{M}(\mathbf{q})$ was the mass matrix, $\mathbf{C}(\mathbf{q},\dot{\mathbf{q}})$ contained the Coriolis forces, $\mathbf{G}(\mathbf{q})$ contained the gravity forces, and $\mathbf{J_c}$ was the Jacobian of the contact forces $\mathbf{F_c}$. $\boldsymbol{\tau}$ was the sum of active joint torques generated by the muscles $\boldsymbol{\tau}_{mus}$ (Eq. S12),

passive joint torques $\boldsymbol{\tau_{pas}}$ (Eq. S14), and joint torques due to external actuation torques $\boldsymbol{\tau_{ext}}$ (Eq. S15). The talus was assumed to be weightless to save computation within the multibody dynamics. This assumption can be made since the talus is a small body which cannot move independently from the other segments since no MTU is connected to it.

### S2.2 Muscle Dynamics

The model was operated using 92 MTUs (see Table S1). MTUs were modeled as three element Hill-type muscles with a contractile element (CE), a parallel elastic element (PEE), and a series elastic element (SEE). The muscle dynamics were described as a function of the activation state $a$ and the CE length state $s$:

$$\dot{a} - r(n_e)\,(n_e - a) = 0, \tag{S2}$$

$$F_{SEE}(l_{MTU}(\mathbf{q}), s) - (F_{CE}(a, s, \dot{s}) + F_{PEE}(s))\, cos(\phi(s)) = 0, \tag{S3}$$

where the state variable $s = l_{CE}\, cos(\phi)$ denoted the projection of CE length on the muscle line of action for a specific pennation angle $\phi$[3], $\dot{a}$ denoted the time derivative of the activation $a$, $n_e$ denoted the neural excitation, and $r(n_e)$ denoted the activation dynamics, which were determined as follows:

$$r(n_e) = \frac{n_e}{T_{act}} + \frac{1 - n_e}{T_{deact}}. \tag{S4}$$

The activation time constant $T_{act} = 10\,\text{ms}$ and the deactivation time constantand $T_{deact} = 40\,\text{ms}$ were identically to Hamner's model[1]. In Eq. S3, $F_{SEE}$ denoted the force in the SEE, $F_{CE}$ denoted the force in the CE, and $F_{PEE}$ was the force in the PEE. The force in the CE was determined by

$$F_{CE} = a\, f_{FL}(l_{CE})\, f_{FV}(v_{CE})\, F_{ISO}, \tag{S5}$$

where $F_{ISO}$ was the maximum isometric force, $f_{FL}(l_{CE})$ denoted the force-length relationship, and $f_{FV}(v_{CE})$ the force-velocity relationship. The force-length relationship was described as

$$f_{FL}(l_{CE}) = \exp\left(-\left(\frac{l_{CE} - 1}{w}\right)^2\right), \tag{S6}$$

where $l_{CE}$ was the length of the CE normalized to the optimal fibre length $l_{CE,opt}$, $w$ a width parameter of the muscle, equal to the square root of the shape factor used by Thelen[4]. The force-velocity relationship was described as

$$f_{FV}(v_{CE}) = \begin{cases} \frac{\lambda\, v_{CE,max} + v_{CE}}{\lambda\, v_{CE,max} - v_{CE}\, /\, A} & \text{if } v_{CE} < 0 \\ \frac{g_{max}\, v_{CE} + \lambda\, c_{FV}}{v_{CE} + \lambda\, c_{FV}} & \text{if } v_{CE} \geq 0, \end{cases} \tag{S7}$$

where $v_{CE}$ was the normalized CE velocity, $\lambda = 0.5025 + 0.5341a$[5] modeled the activation dependence of the normalized maximum shortening velocity $v_{CE,max} = 10\,\mathrm{l_{CE,opt}/s}$[4]. $g_{max} = 1.8$ was the maximum force amplification during lengthening[4], $A = 0.25$ was the Hill curve parameter[6], and $c_{FV}$ was determined as follows[7]:

$$c_{FV} = \frac{v_{CE,max}\, A\, (g_{max} - 1)}{A + 1}. \tag{S8}$$

The force in the SEE and PEE were modeled as non-linear springs[7]:

$$F(l) = \begin{cases} F_{ISO}\, k_1\, (l - l_{slack}) & \text{if } l \leq l_{slack} \\ F_{ISO}\, k_1\, (l - l_{slack}) + F_{ISO}\, k_2\, (l - l_{slack})^2 & \text{if } l > l_{slack}, \end{cases} \tag{S9}$$

where $l$ denoted the length of the element normalized to optimal fiber length $l_{CE,opt}$. It was equal to $l_{MTU}(\mathbf{q}) - l_{CE}$ for the SEE, and equal to $s$ for the PEE. $l_{slack}$ was the slack length normalized to optimal fiber length $l_{CE,opt}$, $k_1 = 1$ was a small linear stiffness, which was added to aid the optimization such that the derivative with respect to the model states was never zero, and $k_2$ was the non-linear stiffness, which was equal to the following for the PEE and SEE, respectively:

$$k_{2,PEE} = \frac{1}{w^2}, \tag{S10}$$

$$k_{2,SEE} = \frac{1}{(h\, l_{slack,SEE})^2}, \tag{S11}$$

where $h = 0.04$ was the strain of the muscle at isometric force[8,9].

**Table S1.** Abbreviations of muscles used in the model "runMaD".

| Abbreviation | Muscle Name |
|---|---|
| glut_min/med/max | gluteus minimus/medius/maximus |
| semimem | semimembranosus |
| semiten | semitendinosus |
| bifemsh/bifemlh | biceps femoris (short/long head) |
| sar | sartorius |
| add_long/brev/mag | adductor longus/brevis/magnus |
| tfl | tensor fascia latae |
| pect | pectineus |
| grac | gracilis |
| iliacus | iliacus |
| psoas | psoas |
| quad_fem | quadratus femoris |
| gem | gemellus |
| peri | piriformis |
| rect_fem | rectus femoris |
| vas_med/int/lat | vastus medialis/intermedius/lateralis |
| med/lat_gas | gastrocnemius (medial/lateral head) |
| soleus | soleus |
| tip_post/ant | tibialis posterior/anterior |
| flex_dig/hal | flexor digitorum/hallucis |
| per_long/brev/tert | peroneus longus/brevis/tertius |
| ext_dig/hal | extensor digitorum/hallucis |
| ercspn | erector spinae |
| intobl/extobl | internal/external abdominal oblique |

## S2.3 Muscle-Joint Coupling

The torque in DOF $j$ generated by muscle $i$ was determined using the following equation:

$$\tau_{mus,i,j} = -\frac{\partial\, l_{MTU,i}(\mathbf{q})}{\partial\, q_j}\, F_{SEE,i}\, (l_{MTU,i}(\mathbf{q}), l_{CE,i}), \tag{S12}$$

where $l_{MTU,i}(\mathbf{q})$ denoted the normalized muscle-tendon length depending on the current pose defined by the generalized coordinates $\mathbf{q}$, and $\frac{\partial\, l_{MTU,i}(\mathbf{q})}{\partial\, q_j}$ denoted the muscle moment arm. A constant muscle moment arm would not have been accurate enough in the 3D model[3]. Hence, a polynomial function was fitted to describe the muscle-tendon length depending on the joint angles to match the moment arms available in OpenSim, since polynomials have well defined derivatives. The order of the polynomial was chosen such that the root mean square error in the moment arms was less than 5 % of the maximum moment arm for each muscle. The maximum possible order was set to four. Additionally, the range of motion used to determine the polynomial was reduced if the muscles wrapped around the bones incorrectly for large joint angles. Linear interpolation was used outside of this range of motion. The following ranges were used: hip flexion $[-28°, 78°]$, hip adduction $[-13°, 13°]$, hip rotation $[-3°, 18°]$, knee angle $[-118°, 8°]$, ankle angle $[-38°, 38°]$, subtalar angle $[-13°, 13°]$, mtp angle $[-8°, 48°]$, lumbar extension $[-38°, 3°]$, lumbar bending $[-8°, 8°]$, and lumbar rotation $[-18°, 18°]$.

## S2.4 Passive Joint Torques

Passive torques were added to the joint torques when the joint angle was outside of the normal range of motion. These torques were determined as follows:

$$\tau_{pas,out,j} = \begin{cases} K_2\, (q_j\, - q_{j,min})^2 & \text{if } q_j\, <\, q_{j,min} \\ -K_2\, (q_j\, - q_{j,max})^2 & \text{if } q_j\, >\, q_{j,max}\, , \end{cases} \tag{S13}$$

where $K_2 = 5000\,\mathrm{N\,m\,rad^{-2}}$. The range of motions, defined by $q_{j,min}$ and $q_{j,max}$ for the trunk and legs are the same as those used for the muscle moment arms. For the arms, the following ranges were used: arm flexion $[-88°, 88°]$, arm adduction $[-118°, 88°]$, arm rotation $[-88°, 88°]$, elbow flexion $[2°, 148°]$, and pronation/supination $[2°, 148°]$. For numerical reasons, a small stiffness was used for the full range of motion, such that the derivative of the joint moment with respect to the joint angle was never zero:

$$\tau_{pas,j} = \tau_{pas,out,j} - K_1\, (q_j\, -\, q_{j,neutral})\, -\, B\, \dot{q}_j\, , \tag{S14}$$

where the stiffness was $K_1 = 1\,\mathrm{N\,m\,rad^{-1}}$ and the damping was equal to $B = 1\,\mathrm{N\,m\,s\,rad^{-1}}$. $q_{j,neutral}$ was the neutral position of DOF $j$ defined as default values in the OpenSim model file runMaD.osim (see supplementary material at www.simtk.org).

### S2.5 Arm Torques

The DOF $j$ of a arm was directly actuated by the torque

$$\tau_{ext,j} \;=\; m_j\;10\,\mathrm{N\,m}\,, \tag{S15}$$

with torque control $m_j$. For numerical reasons, a scaling was performed to obtain states and controls of same magnitude.

### S2.6 Penetration-Based Ground Contact Model

Eight contact points were used at each foot to describe the contact with the ground. Four contact points were located at the toe segment and four at the calcaneus segment. In the OpenSim model, their location relative to the respective segment origin was defined using marker objects (see runMaD.osim). The vertical ground reaction force (GRF) in each contact point $c$ was determined based on the ground penetration $d$:

$$F_{c,y}(d) \;=\; k\,d\,(1\,-\,b\,\dot{p}_{c,y})\,, \tag{S16}$$

where $k = 100\,\mathrm{BW\,m^{-1}}$ was the stiffness of the ground normalized to body weight (BW) per meter. The damping constant $b = 0.75\,\mathrm{s\,m^{-1}}$[10] was multiplied with the vertical velocity of the contact point $\dot{p}_{c,y}$. The ground penetration $d$ was determined from the vertical position of the contact point $p_{c,y}$ and the size of the transition region $p_{c,y,0} = 10^{-3}\,\mathrm{m}$ between contact and no contact:

$$d = \frac{1}{2}\left(\sqrt{p_{c,y}^2\,+\,p_{c,y,0}^2}\,-\,p_{c,y}\right)\,. \tag{S17}$$

The horizontal GRFs in x- and z-directions were determined using a continuous approximation of the Coulomb friction:

$$F_{c,x}(F_{c,y},\dot{p}_{c,x}) \;=\; -\,\mu_k\,F_{c,y}\,\frac{\dot{p}_{c,x}}{\sqrt{\dot{p}_{c,x}^2\,+\,\dot{p}_{c,x,0}^2}}\,, \tag{S18}$$

$$F_{c,z}(F_{c,y},\dot{p}_{c,z}) \;=\; -\,\mu_k\,F_{c,y}\,\frac{\dot{p}_{c,z}}{\sqrt{\dot{p}_{c,z}^2\,+\,\dot{p}_{c,z,0}^2}}\,, \tag{S19}$$

where $\mu_k = 1$ was the kinetic friction coefficient, $\dot{p}_{c,x}$ and $\dot{p}_{c,z}$ were the sliding velocities of the contact point, and $\dot{p}_{c,x,0} = \dot{p}_{c,z,0} = 10^{-2}\,\mathrm{m\,s^{-1}}$ was a small velocity parameter that ensured that the force was differentiable around zero velocity.

## S3 Simulations

The bounds of states $\mathbf{x}$ and controls $\mathbf{u}$ used in the three optimization examples are summarized in Table S2. For standing, smaller ranges were chosen compared to running to avoid the simulation terminating in local optima.

## S4 Results

Pelvis translation and joint moments of straight and curved running are shown in Figs. S1 and S2.

**Table S2.** Bounds used to simulate standing, straight running, and curved running. For straight running, the pelvis position at the first node was fixed to $q_{pel\_tx}[0] = 0$ and $q_{pel\_tz}[0] = 0$. For curved running, the pelvis position at the first node was fixed to $q_{pel\_tx}[0] = -r$ and $q_{pel\_tz}[0] = 0$. $\Delta t$ denotes the duration between two collocation nodes.

| Parameter | Unit | Standing | | Running | |
|---|---|---|---|---|---|
| | | Lower | Upper | Lower | Upper |
| $q_{pelvis\_rotation}$ | degree | 0 | 0 | -90 | 90 |
| $q_{pelvis\_obliquity}$ | degree | -5 | 5 | -90 | 90 |
| $q_{pelvis\_tilt}$ | degree | -30 | 30 | -90 | 90 |
| $q_{pel\_tx}$ | m | 0 | 0 | -5 | 7 |
| $q_{pel\_ty}$ | m | 0.5 | 1.5 | -1 | 2 |
| $q_{pel\_tz}$ | m | 0 | 0 | -3 | 3 |
| $q_{hip\_flexion}$ | degree | -30 | 30 | -120 | 120 |
| $q_{hip\_adduction}$ | degree | -10 | 10 | -120 | 120 |
| $q_{hip\_rotation}$ | degree | -30 | 30 | -120 | 120 |
| $q_{knee\_angle}$ | degree | -30 | 10 | -160 | 10 |
| $q_{ankle\_angle}$ | degree | -30 | 30 | -90 | 90 |
| $q_{subtalar\_angle}$ | degree | -30 | 30 | -90 | 90 |
| $q_{mtp\_angle}$ | degree | -30 | 30 | -90 | 90 |
| $q_{lumbar\_extension}$ | degree | -10 | 10 | -90 | 90 |
| $q_{lumbar\_bending}$ | degree | -10 | 10 | -90 | 90 |
| $q_{lumbar\_rotation}$ | degree | -10 | 10 | -90 | 90 |
| $q_{arm\_flex}$ | degree | -40 | 40 | -40 | 40 |
| $q_{arm\_add}$ | degree | -40 | 40 | -40 | 40 |
| $q_{arm\_rot}$ | degree | -40 | 40 | -40 | 40 |
| $q_{elbow\_flex}$ | degree | 0 | 150 | 0 | 150 |
| $q_{pro\_sup}$ | degree | 0 | 150 | 0 | 150 |
| $\dot{\mathbf{q}}$ | $\mathrm{rad\,s^{-1}}$ or $\mathrm{m\,s^{-1}}$ | -30 | 30 | -30 | 30 |
| $\mathbf{s}$ | - | 0 | 5 | 0 | 5 |
| $\mathbf{a}$ | - | 0 | 5 | 0 | 5 |
| $\mathbf{n_e}$ | - | 0 | 5 | 0 | 5 |
| $\mathbf{m}$ | - | -5 | 5 | -5 | 5 |

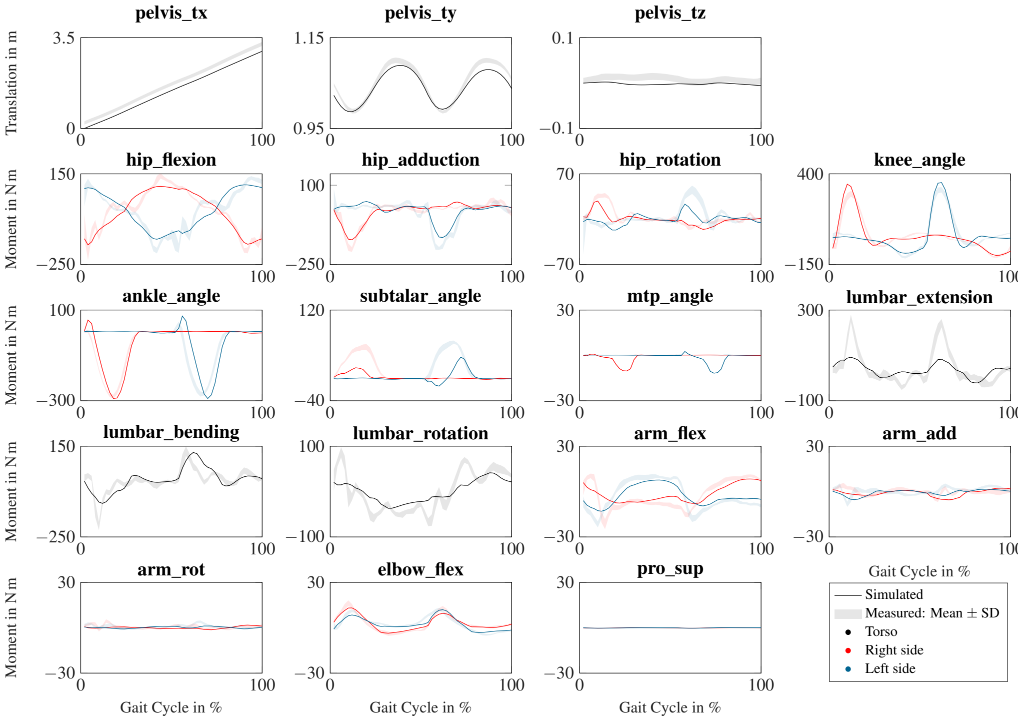


**Figure S1.** Pelvis translation and joint moments of the straight running simulation. The degrees of freedom (DOFs) are named according to their definition in the model file runMaD.osim. Black, red, and blue solid lines indicate the simulated variables of the torso, the right side, and left side, respectively. Shaded areas show mean ± standard deviation (SD) of inverse dynamics (ID) of the measured gait cycles of straight running.

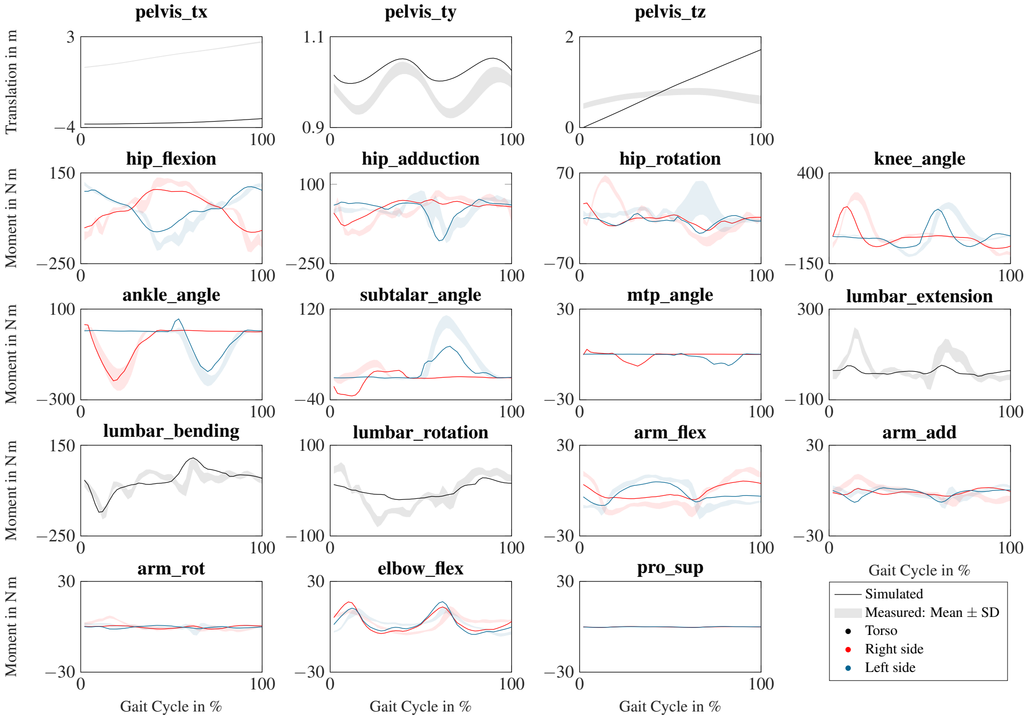


**Figure S2.** Pelvis translation and joint moments of the curved running simulation. The degrees of freedom (DOFs) are named according to their definition in the model file runMaD.osim. Black, red, and blue solid lines indicate the simulated variables of the torso, the right side, and left side, respectively. Shaded areas show mean ± standard deviation (SD) of inverse dynamics (ID) of the measured gait cycles of curved running. The horizontal pelvis translation cannot be directly compared to the measured data since the global frames were not aligned but rotated around the vertical axis.

## References


1. Hamner, S., Seth, A. & Delp, S. L. Muscle contributions to propulsion and support during running. *J. Biomech.* **43**, 2709–2716 (2010).
2. Baker, R. Pelvic angles: a mathematically rigorous definition which is consistent with a conventional clinical understanding of the terms. *Gait & Posture* **13**, 1–6 (2001).
3. Van den Bogert, A. J., Blana, D. & Heinrich, D. Implicit methods for efficient musculoskeletal simulation and optimal control. *Procedia IUTAM* **2**, 297–316 (2011).
4. Thelen, D. G. Adjustment of muscle mechanics model parameters to simulate dynamic contractions in older adults. *J. Biomech. Eng.* **125**, 70 (2003).
5. Chow, J. W. & Darling, W. G. The maximum shortening velocity of muscle should be scaled with activation. *J. Appl. Physiol.* **86**, 1025–1031 (1999).
6. Winters, J. M. An improved muscle-reflex actuator for use in large-scale neuromusculoskeletal models. *Annals Biomed. Eng.* **23**, 359–374 (1995).
7. McLean, S. G., Su, A. & Van den Bogert, A. J. Development and validation of a 3-D model to predict knee joint loading during dynamic movement. *J. biomechanical engineering* **125**, 864–874 (2003).
8. Van Soest, A. J. & Bobbert, M. F. The contribution of muscle properties in the control of explosive movements. *Biol. cybernetics* **69**, 195–204 (1993).
9. Miller, R. H. Hill-based muscle modeling. In *Handbook of Human Motion*, 373–394 (Springer, 2018).
10. Gerritsen, K. G., van den Bogert, A. J. & Nigg, B. M. Direct dynamics simulation of the impact phase in heel-toe running. *J. Biomech.* **28**, 661–668 (1995).
