# Supplementary figures and images for: Efficient trajectory optimization for curved running using a 3D musculoskeletal model with implicit dynamics
Source: Sci Rep. 2020 Oct 19;10:17655. doi: 10.1038/s41598-020-73856-w (PMC7573630; doi:10.1038/s41598-020-73856-w)

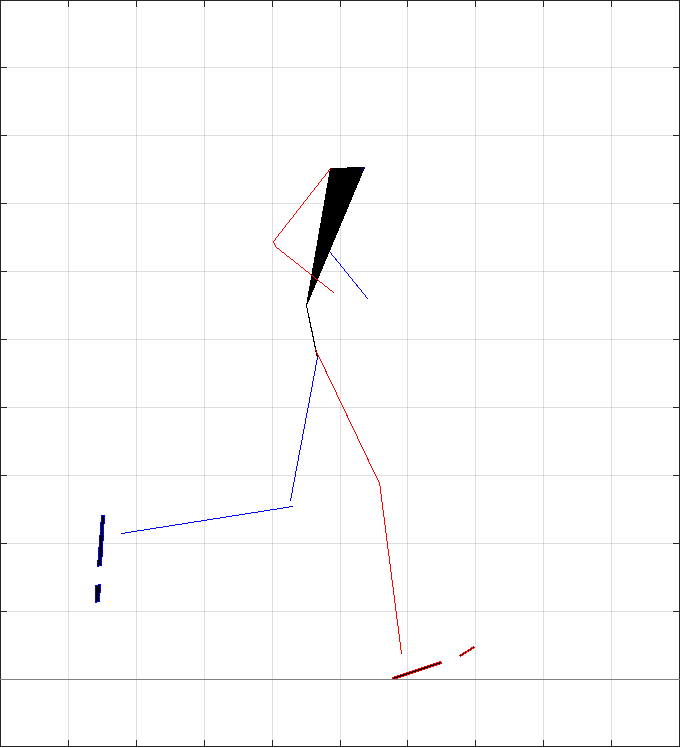

Supplement: Supplementary file 2 — Supplementary information 2. [file 41598_2020_73856_MOESM2_ESM.gif]

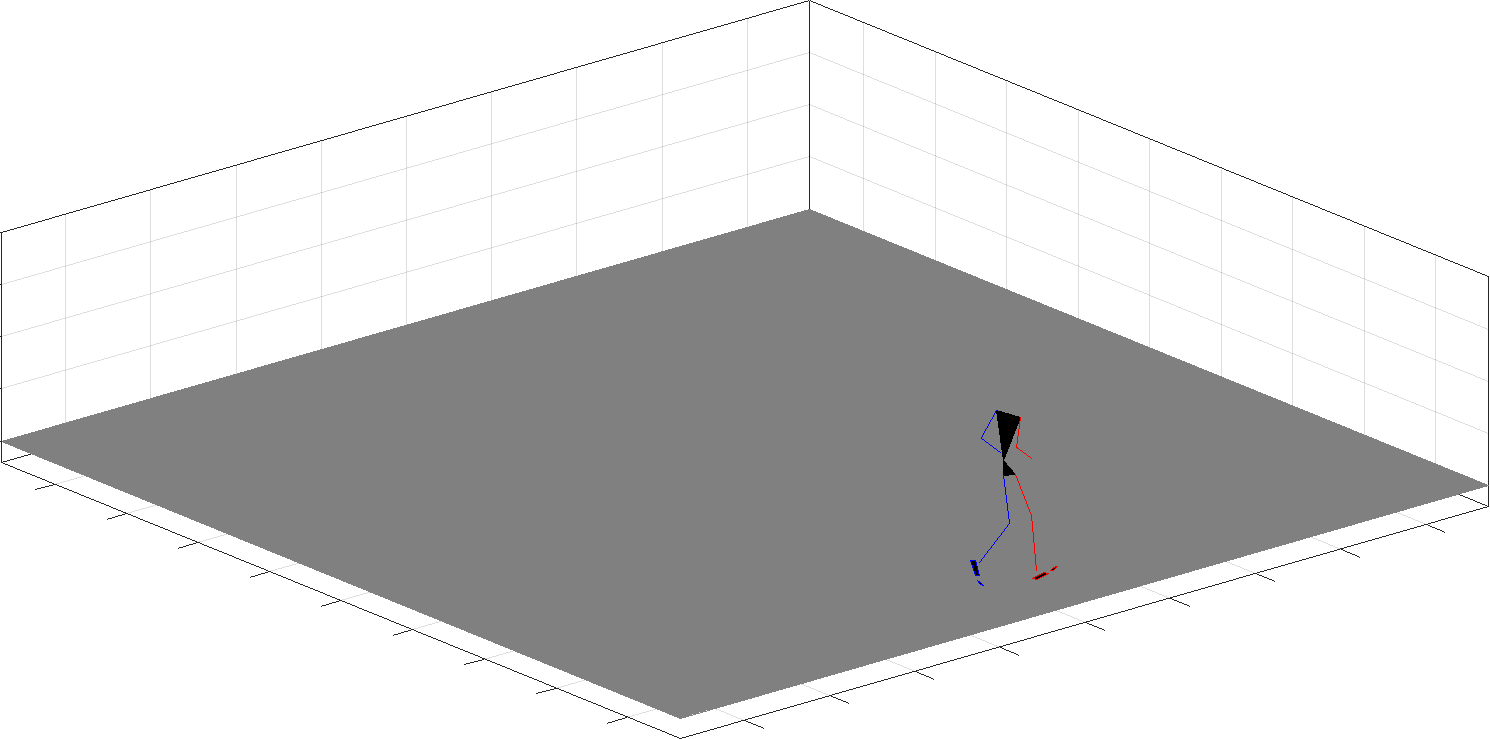

Supplement: Supplementary file 3 — Supplementary information 3. [file 41598_2020_73856_MOESM3_ESM.gif]
